# Circulating MicroRNAs: Potential and Emerging Biomarkers for Diagnosis of Cardiovascular and Cerebrovascular Diseases

**DOI:** 10.1155/2015/730535

**Published:** 2015-05-28

**Authors:** Meng Li, Junping Zhang

**Affiliations:** ^1^Graduate School, Tianjin University of Traditional Chinese Medicine, Tianjin 300193, China; ^2^Department of Cardiology, First Teaching Hospital of Tianjin University of Traditional Chinese Medicine, Tianjin 300193, China

## Abstract

MicroRNAs (miRNAs) are composed of a group of endogenous and noncoding small RNAs which control expression of complementary target mRNAs. The extended functions of miRNAs enhance the complexity of gene-regulatory processes in cardiovascular and cerebrovascular diseases. Indeed, recent studies have shown that miRNAs are closely related to myocardial infarction, heart failure, atrial fibrillation, cardiomyopathy, hypertension, angiogenesis, coronary artery disease, dyslipidaemia, stroke, and so forth. These findings suggest a new therapeutic pointcut for cardiovascular and cerebrovascular diseases and show the extensive therapeutic potential of miRNA regulation. Moreover, it has been shown that circulating extracellular miRNAs are stable in bodily fluids, which indicates circulating miRNAs as potential and emerging biomarkers for noninvasive diagnosis. This review highlights the most recent findings indicative of circulating miRNAs as potential clinical biomarkers for diagnosis of cardiovascular and cerebrovascular diseases.

## 1. Biogenesis of miRNAs 

**Figure 1:**
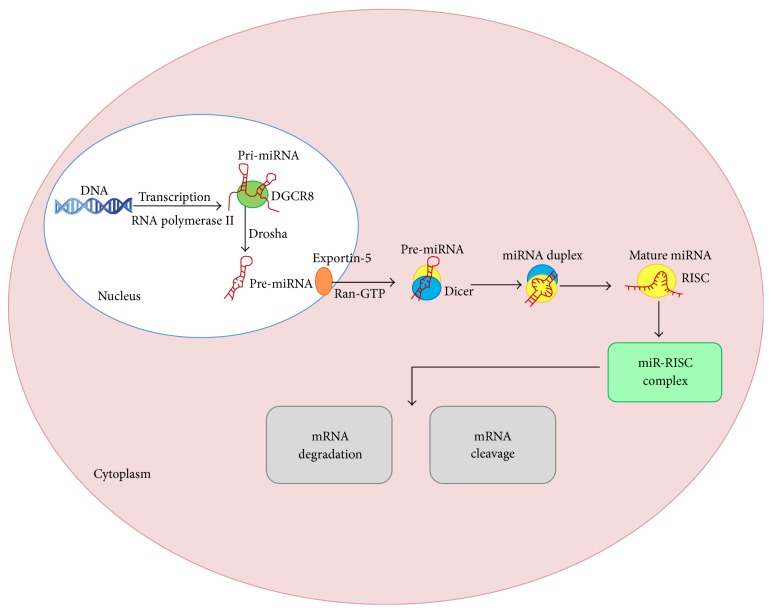
Biogenesis of miRNAs. In the nucleus, miRNAs are transcribed by RNA polymerase II to generate long primary transcripts (pri-miRNAs), which may contain more than one miRNA. Pri-miRNAs are subsequently processed by the RNase III enzyme (Drosha) and its binding partner DGCR8, forming hairpin-like precursor miRNAs (pre-miRNAs). Pre-miRNAs are exported into the cytoplasm by exportin-5 with a Ran-GTP-dependent mechanism. Pre-miRNAs are cleaved by the RNase III enzyme (Dicer) to mature miRNAs. A single strand of the short interfering RNA (siRNA) or miRNA duplex forms RNA-induced silencing complexes (RISC). Then miRNAs guide RISC to complementary sites of the target mRNAs, initiating degradation or cleavage of mRNA.

First discovered in* Caenorhabditis elegans* (*C. elegans*) in 1993, miRNAs inhibited their target genes by mRNA degradation or translational repression [1]. miRNAs are short noncoding RNAs of about 17–25 nucleotides in length. In the nucleus, miRNAs are transcribed by RNA polymerase II to generate long primary transcripts (pri-miRNAs), which may contain more than one miRNA. Pri-miRNAs are subsequently processed by the RNase III enzyme (Drosha) and its binding partner DGCR8, forming hairpin-like precursor miRNAs (pre-miRNAs). Pre-miRNAs are exported into the cytoplasm by exportin-5 with a Ran-GTP-dependent mechanism. Pre-miRNAs are cleaved by the RNase III enzyme (Dicer) to mature miRNAs [2]. A single strand of the short interfering RNA (siRNA) or miRNA duplex forms RNA-induced silencing complexes (RISC). Argonaute (Ago) proteins are the catalytic endonuclease of RISC, and PIWI domain is the catalytic center. Recently, it was found that Dicer and Ago proteins are additional protein cofactors of the holo-RISC complex. The pre-miRNA processing and RISC fitting are functionally coupled, and ATP is not required. Then miRNAs guide RISC to complementary sites (most often located in the 3′-untranslated region) of the target mRNAs, which inhibits the mRNA functions [3, 4] (Figure 1).

## 2. Characteristics and Potential Function of Circulating miRNAs

miRNAs were initially found in intracellular locations, so most studies have assessed the miRNAs expression in original tissue samples. Recently, the discovery of circulating fetal nucleic acids in maternal plasma [5] and detecting circulating extracellular miRNAs in the serum/plasma of cancer patients [6] have suggested a broad opportunity for development of circulating miRNAs as blood-based markers for noninvasive molecular diagnostics. Despite the existence of RNases, miRNAs remain stable in serum and other body fluids [7]. Circulating miRNAs remain stable even after exposure to severe conditions, such as high temperatures, extreme pH, and prolonged storage [8, 9]. Circulating miRNAs are protected themselves from degradation by several mechanisms, including packing in membrane vesicles (such as microvesicles [10, 11], exosomes [12], and apoptotic bodies [13]), bounding to transporter proteins [14], and inclusion in macromolecular complexes (such as high density lipoproteins) [15]. The existence of miRNAs in microvesicles indicates that circulating miRNAs may be a new and potential intercellular communication system which contributes to disease progression [16–18]. Latest study reported that RISC loading complex proteins, Dicer, AGO2, and TRBP, existed in exosomes derived from cancer cells and serum of breast cancer patients. These proteins can process pre-miRNAs into mature miRNAs. Cancer exosomes regulate the nontumorigenic epithelial cells to form tumors. It indicates that miRNAs can manipulate the progression of cancer via exosomes [19]. However, the exact mechanisms of miRNA paracrine signal contributing to disease progression remain to be explored. Circulating miRNAs possess many essential characteristics as good biomarkers: good sensitivity and specificity; noninvasive measurability; long half-life within the samples; and time-related changes during the course of disease [20].

## 3. Detection Method of Circulating miRNAs

Real-time quantitative reverse transcriptase polymerase chain reaction (qRT-PCR) is the most sensitive and specific method applied to quantify circulating miRNAs. Detailed descriptions of this method for miRNAs quantification in serum/plasma are illustrated [21]. The challenges to quantify circulating miRNAs with sufficient sensitivity and precision are as follows: very little amount of RNA recovered from serum or plasma and lack of appropriate endogenous controls for normalization. Because the RNA extracted from plasma/serum is very little, an accurate normalization procedure is necessary. At present, it is possible to normalize the technical variability of the serum/plasma RNA extraction using the* C. elegans* spiked-in control miRNAs. During the RNA extraction, synthetic* C. elegans* miRNAs (miR-54, miR-39) and some other endogenous circulating miRNAs (such as miR-1249, miR-17-5p, U6, miR-454, 5S rRNA, and RNU6b) can be added after denaturation of serum/plasma [22]. It should be noticed that antiplatelet [23] and anticoagulation [24] drugs can affect miRNA quantification in blood samples and must be taken into account when detecting circulating miRNAs. However, real-time PCR based detection of circulating miRNA is ligation based, which potentially creates bias and poses a challenge to quantification.

## 4. Application of Circulating miRNAs in Diagnosis of Cardiovascular and Cerebrovascular Diseases

In the past few years, several studies have reported the use of miRNAs as circulating biomarkers for diagnosis or prognosis of cardiovascular and cerebrovascular diseases, such as myocardial infarction, heart failure, atrial fibrillation, cardiomyopathy, hypertension, coronary artery disease, angiogenesis, dyslipidaemia, and stroke (Table 1).

## 5. Acute Myocardial Infarction

Acute myocardial infarction (AMI) is characterized by cardiac cell death after ischemia. Damaged cells release various proteins into circulation, including cardiac myoglobin, creatine kinase-MB (CK-MB), cardiac troponins I (cTn I), and cardiac troponins T (cTn T), which have been extensively used as standard biomarkers for diagnosis in the clinic [25]. However, the biggest defect of these diagnostic assays of AMI is that some other diseases, such as renal and heart failure, can increase the circulating biomarkers without AMI [26, 27]. Therefore it is necessary to explore novel approaches which can improve and supplement the current strategies for AMI diagnosis.

AMI induces the cardiac-specific miRNAs released from injured cardiomyocytes into circulation. The plasma miR-208a significantly increased in isoproterenol-induced myocardial injury rat models and had a good correlation with cTnI. To exclude the possibility that the increased plasma miR-208a was caused by nonspecific injury, it was also examined in a renal infarction model [28]. The result showed that the plasma miR-208a remained undetectable, which indicated that the circulating miR-208a had specificity in AMI prediction. In clinical trial the cardiac-specific miR-208a increased in AMI patients with a detection sensitivity of 90.9% but was undetectable in healthy controls, which was consistent with animal experiments. A parallel analysis of circulating miR-208a and cTnI showed miR-208a was increased in all AMI individuals but cTnI was detectable only in 85% of patients within 4 hours of the symptoms onset [29]. Moreover, in end-stage renal diseases, the cTnI and cTnT increased occasionally without AMI [28]. Therefore, circulating miR-208a may be alternative or even superior to conventional biomarkers (cTnI and cTnT) for the early detection of AMI. However, several researches showed the circulating miR-208a was in very low levels or could not be detected in AMI patients [30]. The inconsistency is probably related to the sampling time. The miR-208a peaks 3 hours after AMI and returns to baseline after 24 hours [31]. It was also reported that the circulating miR-1 significantly increased in AMI patients compared to non-AMI controls. Circulating miR-1 was correlated with abnormal QRS widening in AMI patients, whereas no correlation was found with ST-segment alterations [32]. However the relevance between miR-1 and conventional biomarkers (cTnI, cTnT, and CK-MB) remains controversial [30, 33]. Additional miRNAs that were found to be increased in AMI patients include miR-133a [34], miR-133b [35], miR-499 [36], miR-499-5p [37], miR-328 [38], miR-1291, and miR-663b [39], whereas miR-223 [40], miR-122, and miR-375 [30] were decreased. In addition, a prospective research indicated that circulating miR-197 and miR-223 had negative correlations with AMI incidence. On the contrary, circulating miR-126 showed a positive correlation with AMI incidence [41].

## 6. Heart Failure

Clinical management of heart failure (HF) is facilitated by circulating biomarkers, such as brain natriuretic peptide (BNP) and N-terminal pro-brain-natriuretic-peptide (NT-pro-BNP) [42]. However, it is still necessary to find a more reliable and objective measurement for HF diagnosis and management. It has been reported that patients recruited from a dyspnea registry were distinguished between dyspnea due to HF and dyspnea without HF by circulating miR-423-5p. Circulating miRNA-423-5p also correlated with NT-pro BNP [43]. In addition, meticulous screening of 186 miRNAs uncovered four main miRNAs (miR-423-5p, miR-22, miR-320a, and miR-92b) significantly increased in the serum of HF patients. With the detection of the four miRNAs, a sensitive and specific score could be defined for assessing HF patients. The miRNA-score was closely related to several important prognostic parameters, including increased serum BNP, widening QRS, and dilatation of left ventricle and atrium [44]. Another research found circulating miR-499 significantly increased in patients with acute heart failure [45].

## 7. Atrial Fibrillation

Many biomarkers have been assessed for their correlation with atrial fibrillation (AF). However, the relative stability of circulating miRNAs and the roles miRNAs play in AF indicated that circulating miRNAs might be potential biomarkers for AF diagnosis. Massively parallel signature sequencing was used to carry out an in-depth analysis of the miRNA expression profile in 5 healthy controls, 5 patients only with paroxysmal atrial fibrillation (PAF), and 5 patients only with persistent atrial fibrillation (PersAF). Twenty-two specific miRNAs were found in each group. Four candidate miRNAs (miRNA-146a, miRNA-150, miRNA-19a, and miRNA-375) met the choice criteria and were detected in an independent cohort of 90 plasma samples by TaqMan miRNA qRT-PCR. It finally proved that plasma miRNA-150 levels decreased significantly in AF patients [46].

## 8. Cardiomyopathy

Several researches have demonstrated a functional role of miRNAs in cardiomyopathy. Hypertrophic cardiomyopathy (HCM) is an inherited heart disease with a prevalence of approximately 1 : 500 among the general population. The study aiming to characterize the circulating miRNA profile of HCM enrolled forty-one HCM patients who were characterized with conventional transthoracic echocardiography and cardiac magnetic resonance, and 41 age- and sex-matched healthy people were as control. The result showed 12 miRNAs significantly increased in plasma of HCM patients. However, correlation with left ventricular hypertrophy parameters held true for only 3 miRNAs (miR-199a-5p, miR-27a, and miR-29a), whereas only miR-29a was significantly associated with both hypertrophy and fibrosis. So miR-29a was identified as a potential biomarker for HCM assessment [47]. Another research was about dilated cardiomyopathy (DCM). It reported that the miRNA expression profiles were examined in stable patients with isolated diastolic dysfunction, patients with stable compensated DCM, and those with decompensated congestive heart failure secondary to DCM (DCM-CHF). MiR-142-3p decreased in DCM and DCM-CHF groups and miR-124-5p only increased in DCM group [48]. Takotsubo cardiomyopathy (TTC) is an increasingly recognized acute syndrome with similar symptoms to AMI. The TTC symptoms include chest pain and electrocardiographic changes, mainly in the absence of obstructive coronary artery disease. The study showed a unique signature comprising miR-1, miR-16, miR-26a, and miR-133a differentiated TTC from healthy people and ST-segment elevation acute myocardial infarction (STEMI), which has an important meaning for diagnosis [49].

## 9. Hypertension

Hypertension is an epidemic condition. Approximately 90–95% of hypertension is the essential hypertension subtype. One study demonstrated a novel link between human cytomegalovirus (HCMV) infection and essential hypertension. It showed that 27 differentially expressed circulating miRNAs were identified in 13 essential hypertensive patients and 5 healthy control subjects. The expressions of miR-296-5p, miR-let-7e, and hcmv-miR-UL112 (a human cytomegalovirus-encoded miRNA) were validated independently in plasma samples [50]. Another study detected and analyzed plasma samples from three independent cohorts to identify circulating miRNAs candidates in essential hypertension patients. The results indicated that the plasma hsa-miR-505 was significantly elevated in essential hypertensive patients. The circulating hsa-miR-505 may be a novel circulating signature of hypertension [51]. Recently, more and more researches focus on the association between circulating miRNAs and pulmonary. A total of 40 human subjects were included in the study and the degree of pulmonary hypertension (PH) was determined by the mean pulmonary arterial pressure. It identified several novel upregulated miRNAs (miR-23b, miR-130a, and miR-191) and downregulated miRNAs (miR-451, miR-1246) in the circulation of PH subjects. It indicated that miRNAs may be considered as potential biomarkers for early diagnosis of PH [52]. One study conducted miRNA profiles of plasma from PH patients and PH rats induced by monocrotaline. The result showed miR-26a decreased in both experimental and clinical PH, and it could be a robust marker and intervention target of PH [53]. Another research also found that reduced miRNA-150 was closely associated with poor survival in PH patients [54].

## 10. Coronary Artery Disease

Coronary artery disease (CAD) is a major cause of death and disability in developed countries and some developing countries. Atherosclerosis is the main cause of CAD. It is characterized by endothelial activation, plaque formation, and structural remodeling of arterial wall. To identify unstable plaques has a great clinical meaning for prevention and treatment of acute coronary syndromes. Currently, fibrinogen and C-reactive protein have been used as CAD markers. However, there still exists limitation of these markers in the diagnosis of cardiovascular conditions, because they can be affected by CAD-unrelated environmental factors and disease backgrounds [55]. Moreover, the available imaging techniques have limited value for the early diagnose of CAD. So it is necessary to find effective and specific biomarkers to evaluate plaque stability and atherosclerosis.

It has been reported that plasma levels of endothelial cell enriched miRNAs (miR-126, miR-92a, and miR-17), smooth muscle-enriched miR-145, and inflammation associated miR-155 significantly reduced in CAD patients compared with healthy controls [31]. Another research detected 157 different miRNAs in PBMCs of CAD patients by miRNA microarray. The result showed that miR-135a increased and miR-147 decreased significantly in plasma of CAD patients, and miR-135a/miR-147 ratio could be used for CAD diagnosis. It also indicated that patients with unstable angina could be differentiated from patients with stable angina by their increased level of miR-134, miR-198, and miR-370, which suggested circulating miRNAs could be used to identify patients at risk for acute coronary syndromes [56]. Furthermore, miR-149 was closely associated with increased risk for CAD in Chinese Han people [57]. Serum miR-31 is higher in CAD patients with restenosis compared to CAD patients without restenosis [58]. Circulating miR-133a and miR-208a levels were upregulated while miR-126, miR-17, miR-92a, and miR-155 levels were significantly downregulated in patients with stable coronary artery disease compared with healthy controls [31]. miR-214 was beneficial for CAD patients, which might be a promising biomarker for alerting severe CAD. Loss of its protection might lead to increased level of placental growth factor and worsening atherosclerosis [59]. Recently, a study revealed increased circulating miR-122 and miR-370 might be associated with the presence as well as the severity of CAD in hyperlipidemia patients. The levels of miR-122 and miR-370 were also positively correlated with TC, TG, and LDL-C levels [60]. All in all, circulating miRNAs have potential to improve the CAD diagnosis.

## 11. Stroke

Stroke is a multifactorial disease with a short therapeutic window. It is one of the main causes of death and disability throughout the world. However, the clinical methods available for the diagnosis and prognosis of stroke are limited to radiological imaging, which is with limited availability and higher cost. Given the limited recommended therapeutic window for thrombolysis (even 4.5 hours after the onset of symptoms) [61], new biomarkers for expediting diagnosis of stroke are necessary. Several studies have found circulating miRNAs change following stroke in both animal models and human.

In animal experiments, initial study indicated circulating miR-124 (a brain-specific miRNA) can be a potential biomarker for diagnosis of cerebral ischemia in rats induced by middle cerebral artery occlusion (MCAO). The plasma miR-124 levels significantly increased after MCAO and peaked at 24 hours (up to 150-fold compared to sham-operated controls), which demonstrated the potential of brain-specific miRNAs to serve as biomarkers of tissue injury [62]. A related study measured an increased plasma miR-124 as early as 6 hours following reperfusion. However, both of the studies did not investigate the changes of miRNA profile in the period immediately following ischemia (before reperfusion) [63]. With the similar animal models of stroke, the studies showed increased circulating miR-125b-2^*∗*^, miR-27a^*∗*^, miR-422a, miR-488, and miR-627 could reflect the onset of ischemic stroke and prove to be of diagnostic value [64]. miR-290 elevated at 24 hours after reperfusion [65]. miR-10a, miR-182, miR-200b, and miR-298 increased in both blood and brain 24 hours following ischemia/reperfusion [66].

In human subjects, testing circulating miRNAs by miRNA microarray and real-time PCR analyses showed that increased hsa-miR-106b-5P and hsa-miR-4306 and decreased hsa-miR-320e and hsa-miR-320d in plasma might be novel biomarkers for the early detection of acute stroke in humans [67]. Another study aimed to explore the possible associations between circulating miRNAs and stroke severity and their involvement in the regulation of inflammatory responses after stroke. The result showed that serum miR-124, miR-9, and miR-219 are suppressed in acute ischemic stroke, which facilitated neuroinflammation and brain injury [68]. It also reported that circulating miR-30a and miR-126 levels were markedly downregulated in all patients with ischemic stroke until 24 weeks. Circulating let-7b decreased in patients with large-vessel atherosclerosis than healthy controls, whereas it increased in patients with other kinds of ischemic stroke until 24 weeks. These circulating miRNAs returned to normal 48 weeks after symptom onset. However, the different expression of let-7b in various types of ischemic stroke deserves further investigation [69]. In patients with acute ischemic stroke, miR-122, miR-148a, let-7i, miR-19a, miR-320d, and miR-4429 decreased and miR-363 and miR-487b increased compared to vascular risk factor controls. These miRNAs may regulate leukocyte gene expression in ischemic stroke including pathways involved in immune activation, leukocyte extravasation, and thrombosis [70]. Detecting serum miR-210 in stroke patients in 3, 7, and 14 days following stroke, it showed the decrease of miR-210 was associated with poor clinical outcome [71]. These studies collectively suggest that miRNA profile changes in both brain and peripheral blood. However a further exploration is necessary to clarify the time course of expression and to correlate miRNA changes with the severity of stroke.

## 12. Limitations and Future Directions

Circulating miRNAs or miRNA combinations can be used as potential biomarkers for diagnosis and prognosis of cardiovascular and cerebrovascular diseases. However there still exist several problems. Firstly, the samples of the studies aimed to identify circulating miRNAs as biomarkers of cardiovascular and cerebrovascular diseases are relatively small. The conclusions should be validated in independent and large cohort studies. Secondly, the expression profile of circulating miRNAs may change depending on the disease state, which makes it difficult to determine appropriate endogenous controls. Thirdly, RNA isolation from blood and subsequent quantification by real-time PCR consume time. So it is necessary to find a better method to detect circulating miRNAs, which makes it available in clinic. With development of high-throughput platforms including multiplex PCR and microarrays, the next-generation-sequencing technology has quickly emerged as the preferred platform for studying circulating miRNAs. The next-generation-sequencing technology has the ability to pool and sequence multiple samples in one lane of a sequencer, which makes it possible to construct comprehensive expression profiles for every assessed sample and lower the costs significantly [72]. In the future, circulating miRNAs may be extensively used in clinical diagnosis. Moreover, it has potential to detect and predict the therapeutic effect of cardiovascular and cerebrovascular diseases.

## Figures and Tables

**Table 1:** Overview of circulating miRNAs in various cardiovascular and cerebrovascular diseases.

Diseases type	miRNAs	Relationship	References
AMI	miR-208a	+	[28, 29, 31]
	miR-1	+	[32]
	miR-133a	+	[34]
	miR-133b	+	[35]
	miR-499	+	[36]
	miR-499-5p	+	[37]
	miR-328	+	[38]
	miR-1291, miR-663b	+	[39]
	miR-223	−	[40]
	miR-122, miR-375	−	[30]
	miR-197, miR-223,	−	[41]
	miR-126	+	[41]

HF	miRNA-423-5p	+	[43]
	miR-423-5p, miR-22, miR-320a, miR-92b	+	[44]
	miR-499	+	[45]

AF	miRNA-150	−	[46]

HCM	miR-29a	+	[47]

DCM	MiR-142-3p	−	[48]
	miR-124-5p	+	[48]

TTC	miR-1, miR-16, miR-26a, miR-133a	+	[49]

Hypertension	miR-296-5p, miR-let-7e, hcmv-miR-UL112	+	[50]
	hsa-miR-505	+	[51]
	miR-23b, miR-130a, miR-191	+	[52]
	miR-451, miR-1246	−	[52]
	miR-26a	−	[53]
	miRNA-150	−	[54]

CAD	miR-126, miR-92a, miR-17, miR-145, miR-155	−	[31]
	miR-135a	+	[56]
	miR-147	−	[56]

CAD (differentiate unstable angina from stable angina)	miR-134, miR-198, miR-370	+	[56]

CAD	miR-149	+	[57]

CAD (with restenosis)	miR-31	+	[58]

CAD	miR-133a, miR-208a	+	[31]
	miR-126, miR-17, miR-92a, miR-155	−	[31]
	miR-214	−	[59]
	miR-122, miR-370	+	[60]

Stroke	miR-124	+	[62, 63]
	miR-125b-2^*∗*^, miR-27a^*∗*^, miR-422a, miR-488, miR-627	+	[64]
	MiR-290	+	[65]
	MiR-10a, miR-182, miR-200b, miR-298	+	[66]
	hsa-miR-106b-5P, hsa-miR-4306	+	[67]
	hsa-miR-320e, hsa-miR-320d	−	[67]
	miR-124, miR-9, miR-219	−	[68]
	miR-30a, miR-126	−	[69]
	miR-122, miR-148a, let-7i, miR-19a, miR-320d, miR-4429	−	[70]
	miR-363, miR-487b	+	[70]
	miR-210	−	[71]

AMI indicates acute myocardial infarction; HF, heart failure; AF, atrial fibrillation; HCM, hypertrophic cardiomyopathy; DCM, dilated cardiomyopathy; TTC, Takotsubo cardiomyopathy; CAD, coronary artery disease.
